# Research on the mechanisms of plant bioactive metabolites in anti-skin aging and future development prospects

**DOI:** 10.3389/fphar.2025.1673075

**Published:** 2025-10-09

**Authors:** Haoran Wang, Jinyv Zheng, Shikui Cao, Jingwei Lv

**Affiliations:** ^1^ College of Clinical Medicine, Changchun University of Chinese Medicine, Changchun, China; ^2^ School of Pharmaceutical Sciences, Changchun University of Chinese Medicine, Changchun, China; ^3^ Graduate School, Changchun University of Chinese Medicine, Changchun, China

**Keywords:** skin aging, plant bioactive metabolites, antioxidant, inflammation, reactive oxygen species (ROS)

## Abstract

With the accelerated pace of modern life and the influence of various environmental factors, skin aging has become a widespread health and aesthetic concern worldwide. Therefore, there is an urgent need for safe, effective and cost-efficient options for the prevention and treatment of skin aging. Researchers have been actively exploring anti-skin aging metabolites that can replace synthetic chemical agents. In recent years, natural plant bioactive metabolites isolated from plants have been considered as good alternatives and have been favored by an increasing number of researchers due to their rich content of bioactive metabolites and low side effects. Botanical bioactive metabolites have become a focal point in the field of anti-aging skincare due to their effectiveness in alleviating visible signs of skin aging and their potential to enhance the overall health of the skin. We collected relevant literature published between 2010 and 2025 using keywords such as “skin aging,” “plant bioactive metabolites,” “antioxidant,” “reactive oxygen species,” “matrix metalloproteinases,” “inflammation,” and others. This review describes skin aging, common plant (e.g., rice, ginseng, tea, etc.) bioactive metabolites and their efficacy and mechanism of action in delaying skin aging. In addition, the development potential and future trends of botanical bioactive metabolites are explored with the aim of providing a more holistic approach to skin aging care and offering valuable insights into the use of botanical bioactive metabolites as important metabolites in the formulation of anti-aging products.

## 1 Introduction

Skin aging is a complex biological process driven by a combination of intrinsic (e.g., genetics, hormones) and extrinsic (e.g., UV rays, pollution) factors, which manifests itself in features such as wrinkles, laxity, and pigmentation, and has become a health and aesthetic issue of global concern (Zhang et al., 2021; Franco et al., 2022).

In recent years, women, especially in the 31–40 age group, have been urgently seeking care and treatment for skin aging (Almohideb, 2021). Previously, a variety of methods were used to combat skin aging, including plastic surgery, the use of chemically synthesised products such as sunscreens (Ganceviciene et al., 2012), and the maintenance of good lifestyle habits (Millar, 2018). Currently, many natural metabolites have been found to possess a variety of biochemical properties such as anti-aging, antioxidant, anti-inflammatory, and antibacterial properties (Papaemmanouil et al., 2022). These benefits stem from the fact that natural metabolites often contain a variety of bioactive metabolites that can enhance the health and appearance of the skin, with plant bioactive metabolites being the main source (Pintus et al., 2022). Therefore, the exploration of natural plant bioactive metabolites that are gentle, have few side effects, and are capable of combating skin aging has become a direction of development in dermatology, skincare, cosmetology, and other related disciplines. The use of plant bioactive metabolites in skin care is often supported by traditional knowledge and practices, which can provide which lends credibility to their efficacy. Many cultures have long utilized specific plants for skin benefits and modern research is beginning to validate these traditional uses (Ceccacci et al., 2022). There are now studies demonstrating that plant bioactive metabolites can stop and improve skin aging (Son et al., 2020).

The use of plant bioactive metabolites in anti-aging products offers several advantages over traditional synthetic products. Firstly, there is a growing demand for natural and sustainable metabolites in today’s society, and the production of plant bioactive metabolites from renewable resources has a lower environmental impact than metabolites that may involve harmful chemicals and processes (Carvalho et al., 2023; Pham et al., 2022), which is in line with consumers' preference for products that are both effective and environmentally responsible. Second, anti-aging products derived from nature also provide consumers with a sense of security (Pham et al., 2022). The gentleness of plant-based formulations can lead to better tolerability and fewer side effects. Consumers with sensitive skin types may have adverse reactions to harsh chemicals commonly found in traditional anti-aging products (Jaros-Sajda et al., 2024). Botanical bioactive metabolites are biocompatible and reduce the risk of skin irritation compared to traditional skin care product metabolites, which is especially beneficial for sensitive skin types. In conclusion, anti-aging products utilizing plant bioactive metabolites offer a range of advantages over conventional products, particularly their ability to provide natural antioxidant and anti-inflammatory benefits, inhibit skin aging-related enzymes and reduce the risk of irritation. The shift from traditional synthetic products to those using plant bioactive metabolites holds great promise for the development of effective anti-aging products.

## 2 Skin aging

**FIGURE 1:**
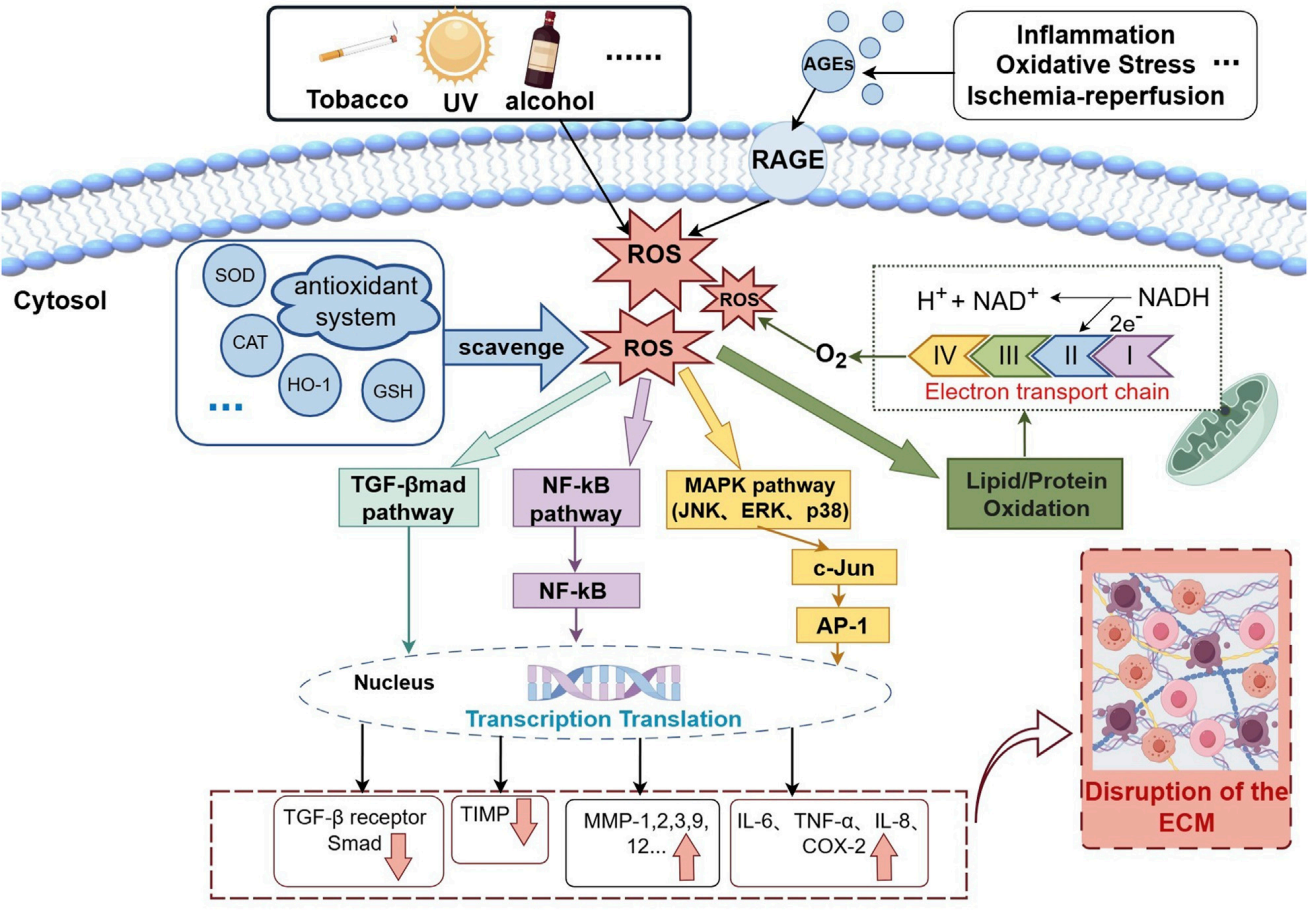
Schematic diagram of intrinsic/extrinsic factor-mediated skin aging. Intrinsic factors such as mitochondrial dysfunction, inflammation, etc. and extrinsic factors such as UV radiation generate ROS. when excessive ROS are generated, they exceed the ability of the body’s antioxidant system to eliminate ROS, and directly damage lipids, proteins, etc. ROS can also activate the MAPK and NF-κB signal pathways, leading to the activation of AP-1 and NF-κB. They can increase the expression of pro-inflammatory cytokines such as IL-8, TNF-α, IL-6, COX-2, etc. to regulate the inflammatory response, and by activating matrix metalloproteinases such as MMP-1, 2, 3, etc., they can reduce the expression of TIMPs, causing an imbalance in the ratio of MMPs/TIMPs, thus rupturing the ECM. At the same time, they can regulate the TGF-β/Smad signal pathway, which ultimately accelerates Skin aging.

Intrinsic/extrinsic factors play an important role in the skin aging process (Figure 1). Extrinsic aging, also known as environmental aging, is mainly the effect of environmental factors (e.g., sunlight exposure (Wong and Chew, 2021; Tai et al., 2024; Flood et al., 2019), air pollution (Wong and Chew, 2021; Liu et al., 2022; Martic et al., 2022; Claros et al., 2023), smoke, etc.) on the skin, which accelerates aging by inducing reactive oxygen species (ROS) generation in large amounts, destroying extracellular matrix (ECM) (e.g., collagen degradation), and triggering inflammatory responses (Wong and Chew, 2021; Tai et al., 2024; Flood et al., 2019; Russell-Goldman and Murphy, 2020; Pourzand et al., 2022). Air pollutants (e.g., exposed chemicals (Mousavi et al., 2021), TCPP (Liu et al., 2022), DPE (Shin et al., 2022), CO (Huang et al., 2022), PM2.5 (Huang et al., 2022), etc.) can have a synergistic effect with UV and exacerbate oxidative damage (Martic et al., 2022; Choi et al., 2023).

Intrinsic aging, also known as physiological aging, involves genetic factors, changes in hormone (e.g., estrogen, thyroid hormone, etc.) levels (Chaudhary et al., 2020; Zhang Z. et al., 2024; Zouboulis and Makrantonaki, 2012). The intrinsic aging process of the skin also involves the action of multiple cell signalling pathways (e.g., p53 and p16INK4a (Shin et al., 2023), GPCR signalling pathway (Cheung et al., 2024)). Therefore, upregulation of certain pathways (e.g., TGF-β/Smad signalling pathway (Zhang and Duan, 2018)) can prevent wrinkles and sagging of skin structures.

## 3 Plant bioactive metabolites

Plant bioactive metabolites are substances extracted from different parts of the plant (e.g., whole plant, leaves, stems, bark, roots, flowers, and fruits). Plant bioactive metabolites are used in a variety of forms; some are made into powders or capsules (Sprung et al., 2023), which are convenient for patients to take and ensure dosage accuracy; they can also be used for topical applications, such as creams and oils (Sprung et al., 2023), which are able to act directly on the skin or localized lesions.

Bioactive metabolites contain the active metabolites of plants and are able to be used in a variety of fields such as medicine, food, and skin care products. Many plant bioactive metabolites are used as sources of traditional medicines for the treatment of various diseases (e.g., oral diseases (Palombo, 2011), respiratory infections (Matotoka et al., 2023), diabetes mellitus (Hasnat et al., 2024; Singh et al., 2024), etc.), as anticancer agents (Hasnat et al., 2024; Sruthi et al., 2023), in the treatment of allergic diseases, and as antiviral agents (Sruthi et al., 2023). Plant bioactive metabolites are rich in bioactive metabolites (Pintus et al., 2022) that inhibit the growth of bacteria and fungi, replace some synthetic preservatives, and fulfil consumer demand for healthy and safe food (Silva et al., 2021). Plant bioactive metabolites can also improve the properties of biopolymers, not only enhancing the functionality of the materials but also their environmental friendliness (Kola and Carvalho, 2023). Plant bioactive metabolites are also rich in antioxidant activity (Hasnat et al., 2024; Jafri et al., 2022), neutralizing free radicals and reducing inflammatory responses (Matotoka et al., 2023; Sruthi et al., 2023). Studies have shown that the amount of collagen in the skin decreases by approximately 1%–1.5% per year in people over the age of 40 (Ansaf et al., 2023). Increased collagen significantly improves the moisture and elasticity of the skin (Campos et al., 2023; Pu et al., 2023; Bolke et al., 2019), making it softer and smoother. Plant bioactive metabolites such as Kaempferia parviflora (Klinngam et al., 2022), Sulforaphane (Ko et al., 2020), purple tulip bioactive metabolites (Hajem et al., 2021), Trigonella foenum-graecum L. (Eaknai et al., 2022), and Physalis peruviana fruit (Cicchetti et al., 2018) can increase collagen synthesis and slow down the aging process. Different plant bioactive metabolites exert their anti-skin aging effects through different mechanisms (Table 1).

**TABLE 1:** Anti-aging mechanisms of key plant bioactive metabolites and related references.

Key plant bioactive metabolites	Anti-aging mechanisms	References
Rosarugosides A (2) and D (1)	Inhibits MMP-1, increases collagen	Franco et al. (2022), Kim et al. (2024a)
Lignan	Reduces ROS and inhibits AGEs formation	Ceccacci et al. (2022)
Glycyrrhiza glabra polyphenols	Antioxidant, inhibits MMPs	Damle and Mallya (2016)
Epigallocatechin gallate (EGCG)	Reduction of ROS, inhibition of MMPs (via NF-κB/AP-1/MAPK pathway)	Chen et al. (2017)
Kuding tea polyphenols (KTPs)	Upregulates T-SOD, CAT, inhibits MMP-2	Yi et al. (2019)
Cherry blossom bioactive metabolites (CBE)	Upregulates SOD to protect keratinocytes from UV damage	Wang et al. (2019a)
7,8-Dihydroxyflavone (7,8- DHF)	Upregulates CAT, Mn-SOD, HO-1, inhibits MMP-1 and increases collagen	Choi et al. (2017)
Laminaria polysaccharide (LP)	Upregulates SOD, CAT and inhibits MMP-1	Hu et al. (2016)
*Centella asiatica* callus bioactive metabolites	Upregulates SOD, CAT and inhibits MMP-9	Buranasudja et al. (2021)
Proanthocyanidins	Scavenges free radicals	Rodríguez-Yoldi (2021)
Rosemarinic acid	Reduction of ROS, anti-inflammatory	Zhu et al. (2022)
Polygonum multiflorum (PMRP)	Improves mitochondrial function, reduces ROS, increases ATP	Liu et al. (2021)
Poria cocos bioactive metabolites	Inhibits MMP-1, increases collagen, and regulates TGF-β/Smad	Fang et al. (2021)
Red ginseng bioactive metabolites	Inhibits MMPs, increases collagen, promotes autophagy	Kim et al. (2020)
Anti-inflammatory phenolics	Inhibits tyrosinase, MMP-2, antioxidant	Emanuele et al. (2017)
Plant-derived extracellular vesicles (EVs)	Downregulates TNF-α, MAPK and NF-κB and Inhibits MMPs	Seyeon et al. (2024)
Flavonoids	Inhibits MMP-1, hyaluronidase	Chaiyana et al. (2020)
Monascus-fermented soybean bioactive metabolites (MFSEs)	Inhibits tyrosinase, hyaluronidase	Jin and Pyo (2017)
1,3,5,6-tetrahydroxyxanthone-C-4-β-d-glucopyranoside	Inhibits tyrosinase, hyaluronidase	El-Nashar et al. (2022)

In recent years, the introduction of nanotechnology and advanced formulation strategies has significantly improved the delivery efficiency and therapeutic efficacy of plant bioactive metabolites (Ahmed et al., 2021). Firstly, the development of synergistic therapeutic strategies between natural active metabolites and synthetic nanomaterials has provided a new direction for the field. A nanofibrous scaffold (NanoPCL-M) based on myrtle bioactive metabolites and polycaprolactone was able to significantly inhibit skin photoaging by activating the regeneration-related pathway of UV-irradiated stem cells, showing good potential for translational applications (Bellu et al., 2020). Secondly, nano-encapsulation technology effectively improves the bioavailability of insoluble plant bioactive metabolites. Encapsulation of curcumin, resveratrol, etc. in liposomes, polymer nanoparticles or nanoemulsions can increase their oral bioavailability by several folds, thus enhancing their anti-inflammatory and antioxidant properties (Karnwal et al., 2024; Dewi et al., 2022). At the same time, nanocarriers excel in protecting sensitive metabolites and improving their stability, with liposomal or polymeric nanoparticles effectively slowing down the oxidation process of antioxidant metabolites (Awlqadr et al., 2025). In addition, lipid nanocarriers (e.g., solid lipid nanoparticles and nanostructured lipid carriers) can effectively load hydrophobic plant bioactive metabolites and improve their solubility and absorption efficiency (Sanjai et al., 2024). At the same time, nanostructures can further reduce enzymatic or pH-sensitive degradation through surface modification (e.g., polyethylene glycolisation), extending the storage life and effectiveness of the active metabolite (Kumari et al., 2022). In addition, green synthesis methods provide new avenues for nanoparticle preparation. Metal nanoparticles (e.g., gold and silver nanoparticles) with good biocompatibility and degradability can be synthesised by using polyphenols, flavonoids and other metabolites in plant bioactive metabolites as reducing and stabilising agents, providing a new approach to the development of plant nanomedicine (Karnwal et al., 2024).

Currently, studies on nanocarrier technology in improving the bioavailability and stability of plant bioactive metabolites are mainly focused on the *in vitro* and animal model stages, and their safety and efficacy have yet to be further verified in clinical trials. In addition, the long-term toxicity, *in vivo* metabolic pathways, and potential effects of nanocarriers on skin microecology have not been clarified. Future studies should pay more attention to the standardised production of nanoformulations, the scale-up preparation process and their actual penetration and mechanism of action in human skin, in order to promote their transformation from laboratory to industrialisation.

## 4 Mechanisms of plant bioactive metabolites against skin aging

### 4.1 Antioxidant activity

Studies have shown that many plant bioactive metabolites have antioxidant metabolites, which are essential for reducing oxidative stress (a key factor in skin aging). It has been found that bioactive metabolites of 16 Thai medicinal plants (e.g., Centella Asiatic, Momordica cochinchinensis, Phyllanthus emblica, etc.) showed significant anti-skin aging effects and were able to improve the structure and function of the skin, which was mainly attributed to their rich content of total phenols and flavonoids (Chaikhong et al., 2023; Poomanee et al., 2023). The bioactive metabolites of Pourthiaea villosa (Thunb.) Decne. bioactive metabolites (PVDE) also contains antioxidant metabolites such as polyols, phenolic and flavonoid metabolites (Choi et al., 2019). Citrus auranticum and Glycyrrhiza glabra are rich in antioxidant polyphenols, which help to prevent skin aging (Damle and Mallya, 2016). Resveratrol-enriched rice DJ526 can be bioactive metabolitesed to resveratrol, a potent antioxidant that is not present in large quantities, and a relatively large yield can be obtained after treatment by YE (yeast bioactive metabolites) (Kantayos et al., 2022). Epigallocatechin gallate (EGCG) is an antioxidant isolated from tea catechins, and in the study the skin condition of the group given EGCG was better than that of the control group, suggesting that EGCG has an antiaging effect (Chen et al., 2017). Bioactive metabolites of species such as Sideritis scardica and Rosa damascena have been found to have a significant role in the development of antioxidant activity through enhancement of cellular function and prevention of UV-induced damage. UV-induced damage by enhancing cellular function and preventing UV-induced damage have shown promising results in combating oxidative damage and promoting skin health (Sklirou et al., 2021). Citral is the main metabolite of lemongrass (*Cymbopogon citratus*) essential oil (LEO), which has significant antioxidant properties, inhibits oxidative degradation, protects the colour and stability of the essential oil’s metabolites, reduces skin irritation and enhances antimicrobial activity (Tran et al., 2021).

#### 4.1.1 Regulation of antioxidant enzymes

Increasing endogenous antioxidant enzymes is one of the antioxidant mechanisms of plant bioactive metabolites. Kuding tea polyphenols (KTPs) were obtained from the plant by an ion precipitation method, and KTPs were shown to significantly increase serum levels of total superoxide dismutase (T-SOD) and catalase (CAT) by UV-induced experiments in mice (Yi et al., 2019). Cherry blossom bioactive metabolites protected human keratinocytes (HaCaT) from UV-induced oxidative stress by increasing the activities of T-SOD and glutathione peroxidase (Wang Y. et al., 2019). *In vitro* experiments showed increased expression of antioxidant enzymes in human dermal fibroblasts (HDF) after PVDE treatment (Choi et al., 2019).7,8-Dihydroxyflavone (7,8- DHF, 7,8-dihydroxy-2-phenyl-4H-chromen-4-one) is a naturally occurring flavonoid found in plants, and DHF effectively attenuated oxidative stress through the upregulation of CAT, manganese-superoxide dismutase (Mn-SOD), and heme oxygenase-1 (HO-1) in aging skin cells (Choi et al., 2017). Topical application of Laminaria polysaccharide (LP) enhanced the expression of antioxidant enzymes in skin tissues with elevated levels of SOD, CAT and glutathione peroxidase (Hu et al., 2016). Hibiscus sabdariffa L. (HS) has a long history of edible and medicinal uses, and several experiments with bioactive metabolites from it have shown that hibiscus acid maintains higher levels of reduced/oxidised glutathione (GSH/GSSG) in skin cells, thus providing a possible mechanism for hibiscus acid antioxidant. The results obtained by RT-qPCR of *Centella asiatica* callus bioactive metabolites clearly indicated that upregulation of cellular antioxidant enzymes seems to be the main reason for the protective effect of callus bioactive metabolites against oxidative stress (Buranasudja et al., 2021). Scutellaria baicalensis Georgi (SBG), a traditional Chinese medicine widely used in the treatment of hypertension and other diseases, showed elevated SOD after topical application on the skin (Sun et al., 2022). Phellinus linteus (PL) is a typical medicinal fungus, and the results of molecular mechanism studies showed that SBG could increase UV-induced SOD activity (Han et al., 2022). Water lily rhizome bioactive metabolites (WLRE) induced an increase in total glutathione, HO-1, which exerts a protective effect against skin aging (Park et al., 2016). An animal study (SAMP1 mouse model) showed that long-term intake of glucoraphanin-enriched kale (GEK) enhanced antioxidant enzyme activity and collagen production via the TβRII/Smad3 pathway in SAMP1 mice, suggesting that GEK is also valuable in preventing skin aging (Chawalitpong et al., 2019).

#### 4.1.2 Reduction of reactive oxygen species (ROS) levels

Reactive oxygen species (ROS) play an important role in both chronic aging and photoaging (Noh et al., 2016). UVB irradiation induces overproduction of ROS in skin cells (Merecz-Sadowska et al., 2021). Oxidative stress leads to cellular dysfunction and even apoptosis and is considered a key factor in aging (Maldonado et al., 2023). When ROS generation exceeds the scavenging capacity of the endogenous antioxidant system, oxidative stress is triggered, which in turn attacks macromolecules such as DNA, proteins, and lipids within the cell. This cumulative damage not only accelerates the process of cellular aging, manifested in wrinkles, sagging and hyperpigmentation phenotypes, but also induces a persistent inflammatory response, forming a vicious cycle that promotes skin aging (Gu et al., 2020; Papaccio et al., 2022).

At the application level, a variety of plant active metabolites demonstrate anti-aging potential by targeting the ROS pathway. Their mechanisms of action mainly include direct scavenging of ROS, enhancement of intracellular antioxidant defences and inhibition of apoptotic signals in the mitochondrial pathway. Proanthocyanidins in grape seed bioactive metabolites and rosmarinic acid in rosemary bioactive metabolites are effective in scavenging free radicals (Rodríguez-Yoldi, 2021; Zhu et al., 2022). Rutin within the bioflavonoid family and Alchemilla mollis (AM) bioactive metabolites exert protective effects by activating the antioxidant system in a dose-dependent manner. (Choi SJ. et al., 2016; Hwang et al., 2018). Polygonum multiflorum (PMRP) improves mitochondrial function and reduces ROS levels by increasing mitochondrial membrane potential and ATP levels (Liu et al., 2021).

In addition, some of the bioactive metabolites are able to help the skin to finely adapt and resist the overproduction of ROS due to environmental stress. Apple mint (Mentha suaveolens Ehrh.) inhibits heat shock-induced ROS production in anti-heat aging skincare products (Son et al., 2018). WLRE and Houttuynia cordata Thunb. (H. cordata) bioactive metabolites metabolite effectively protects UVB-irradiated skin cells by modulating the ROS scavenging pathway and mitochondrial apoptosis mechanism, showing promising applications in photoprotective products (Park et al., 2016; Mapoung et al., 2021).

In summary, a large number of preclinical studies have revealed the potential of various plant bioactive metabolites to reduce oxidative stress by scavenging ROS and enhancing endogenous antioxidant defences. However, it is important to note that most of these promising results are derived from *in vitro* or animal models, and their efficacy, optimal concentrations, and long-term safety in humans have yet to be confirmed by rigorous randomised controlled clinical trials (RCTs).

### 4.2 Inhibition of aging-related enzymes

Enzymes associated with aging include: collagenase, elastase, tyrosinase, etc. Collagenase is the enzyme that can break down collagen. Studies have shown that collagenase expression levels increase during skin aging, accelerating collagen degradation (Shin J-W. et al., 2019). Fragmentation of collagen increases the level of oxidation within damaged cells, further damaging fibroblasts and exacerbating the skin aging process (Lee et al., 2021a). Elastase is primarily responsible for the breakdown of elastin. Accumulation of elastase in skin fibroblasts by ultraviolet B radiation causes degeneration and/or tortuosity of elastic fibres (Tsukahara et al., 2001), a decrease in elastin content, and a decrease in the elasticity of the skin, resulting in skin laxity and the formation of wrinkles (Baumann et al., 2021). The role of tyrosinase in melanin synthesis makes it a key enzyme in the study of skin aging. Abnormal accumulation of melanin may also lead to discolouration and other skin pigmentation problems, which are signs of aging (Skoczyńska et al., 2017). Plant bioactive metabolites (e.g., Salvia officinalis (Khare et al., 2021), Chia (Salvia hispanica) seed (Aguilar-Toalá and Liceaga, 2020), etc.) have been found to be effective in inhibiting the activity of aging-related enzymes, thereby slowing the skin aging process.

#### 4.2.1 Matrix metalloproteinases (MMPs)

Matrix metalloproteinases (MMPs) break down proteins (including elastin and collagen) in the extracellular matrix (ECM) (Choi S. et al., 2016; Zhang et al., 2019), and the integrity of the ECM is threatened, leading to wrinkles and sagging of the skin (Kim et al., 2023). As collagen fibres break down, the skin is poorly hydrated, manifesting as dryness and roughness (Al-Atif, 2022). The structure of elastin fibres allows the skin to quickly regain its shape after stretching (Wang et al., 2021). MMPs have become an important target for skin anti-aging products, and plant bioactive metabolites are able to inhibit MMPs to a certain extent, thereby protecting collagen and elastin in the skin (Philips et al., 2011).

It has been shown that SBG (Sun et al., 2022), PL (70), AM (Hwang et al., 2018), Apigenin (4′,5,7-trihydroxyflavone) (Choi S. et al., 2016), Hawthorn Polyphenol Bioactive metabolites (Liu et al., 2018), enzyme-modified ginseng (EG) bioactive metabolites (Hwang et al., 2014), Poria cocos bioactive metabolites (Fang et al., 2021), the hot water bioactive metabolites of Rosa rugosa’s flower buds (Franco et al., 2022) (Kim KS. et al., 2024), and Protocatechuic acid (PCA) (Shin et al., 2020) downregulated the MMP-1 expression. In the TNF-α (tumour necrosis factor-α)-induced aging model of Hs68 human dermal fibroblasts, 7,8-DHF (0–10 μM) significantly upregulated type I collagen synthesis and inhibited matrix metalloproteinase-1 (MMP-1) expression in a dose-dependent manner after 18 h of treatment (Choi et al., 2017). LP inhibited MMP-1 expression by preventing oxidative stress and JNK phosphorylation, thereby delaying the breakdown of skin collagen during the aging process (Hu et al., 2016). AGEs resulting in increased matrix MMP-1 gene expression (Yoon et al., 2022). Red ginseng NaturalGEL (RG NGEL) made from RG bioactive metabolites reduced UV-induced levels of MMPs (Kim et al., 2020)and increased type I collagen in human fibroblasts. Cyanobacteria bioactive metabolites inhibited MMPs, thus supporting skin elasticity and firmness. The negative effects of H (2)O (2) on MMP 3 and MMP 12 were significantly reduced when evaluated against a mixture of betaine, pentylene glycol, *Saccharomyces cerevisiae* and Rhodiola rosea root bioactive metabolites (BlendE) (Namkoong et al., 2018). By control, Litchi bioactive metabolites inhibited MMP-2 significantly (p < 0.01) more than standard vitamin C (23.75% ± 2.74% and 10.42% ± 5.91% at 0.05 mg/mL, respectively) (Emanuele et al., 2017). *Centella asiatica* callus bioactive metabolites also inhibited the induction of MMP-9 under H2O2 exposure, suggesting potential anti-skin aging activity of the *C. asiatica* callus (Buranasudja et al., 2021). Syringaresinol (SYR) isolated from ginseng berries possesses a variety of physiological activities, showing antioxidant activity and up-regulating autophagic activity in H (2)O (2)-stimulated HaCaT cells, thereby reducing the expression of MMP-2 and MMP-9, which have been associated with skin aging (Choi et al., 2022). Tissue inhibitors of metalloproteinases (TIMPs) are the major endogenous inhibitors of MMPs activity and play a crucial regulatory role. KTPs upregulate the expression of TIMP-1, TIMP-2, and downregulate MMP-2 and MMP-9, and inhibit UVB-induced skin damage (Yi et al., 2019). The anthocyanin metabolite of Vaccinium uliginosum samples is photoprotective, and oral administration of Vaccinium uliginosum attenuates the gene expression of MMPs and increases the levels of TIMP and antioxidant-related genes (Jo et al., 2020).

Inhibits the effects of MMPs on skin aging by modulating multiple signal pathways. Plant-derived extracellular vesicles (EVs) bioactive metabolitesed from Ecklonia cava can reduce the expression levels of MMPs by inhibiting key signaling pathways such as TNF-α, MAPK, and NF-κB (Seyeon et al., 2024). EGCG reduces the expression of MMPs by modulating nuclear factor kappa B (NF-κB), activator protein 1 (AP-1) and mitogen-activated protein kinases (MAPKs) signal pathways in FDP-stimulated HDFs (Wang L. et al., 2019). ALE inhibits heat shock-treated HDFs triggering the production of MMPs and inhibits mitogen-activated protein kinases (MAPKs) with anti-skin heat aging activity (Son et al., 2018). Rutin, a quercetin glycoside, reduced mRNA expression of MMP-1 (80). HcEA bioactive metabolitesed from Cordyceps sinensis inhibited UVB-irradiated skin aging by modulating the MAPK signal pathway, inhibiting JNK/ERK/c-Jun activation and down-regulating the expression of MMP-1 genes and proteins in human dermal fibroblasts (Mapoung et al., 2021). Dieckol (DK), an algal-derived phenolic metabolite, significantly reduced the expression of pro-inflammatory cytokines and MMPs by modulating the NF-κB, AP-1, and MAPKs signal pathways (Wang et al., 2020). Common active metabolites such as CF (Lyu et al., 2022), Pradosia mutisii (Lorz et al., 2019), and PVDE (Hwang et al., 2018), also modulate the activation of the MAPKs inhibitory signal pathway, effectively blocking MMPs production. γ-Mangosteen fruit is an autophagy enhancer, which can be used to enhance MMP-1, MMP-9 through activation of KEAP1/NRF2 signalling and downregulation of MAPK/AP-1/NF-κB-mediated MMP-1, MMP-9 (Kim CW. et al., 2024).

The bioactive metabolites obtained from different bioactive metabolitesion methods showed different inhibitory effects on MMPs. In a study using *in vitro* enzymatic modelling to assess the anti-skin aging potential of Thunbergia laurifolia Lindl. leaf bioactive metabolites, the researchers prepared two bioactive metabolites using Soxhlet bioactive metabolitesion (SE, with 80% ethanol as solvent) and reflux bioactive metabolitesion with deionised water (RE), respectively. The MMP-1 inhibitory activity was determined by luciferase reaction, and the results showed that the SE bioactive metabolites exhibited comparable inhibitory strengths to the positive control gallic acid, with half-maximum inhibitory concentration (IC_50_) values of 12.0 ± 0.3 mg/cm^3^ and 8.9 ± 0.4 mg/cm^3^, respectively (Chaiyana et al., 2020). White rose petal bioactive metabolitesed by three methods (50% ethanol WRPE-EtOH, enzymatic WRPE-enzyme, and high temperature and pressure WRPE-HTHP) showed anti-skin aging activity in in vitro enzymatic assays. All three bioactive metabolites completely inhibited MMP-1 activity within 60 min at a concentration of 100 μg/mL, but only WRPE-EtOH and WRPE-enzyme partially inhibited it (50%–70%) at 50 μg/mL. All three inhibited elastase at high concentrations (≥250 μg/mL), and only WRPE-EtOH was effective at low concentrations (15.6–125 μg/mL).WRPE-EtOH inhibited tyrosinase best, with 80% inhibition at 125 μg/mL. It indicates that the ethanolic bioactive metabolites of white rose has multi-targeted anti-skin aging potential (Choi et al., 2015).

Current studies have mostly focused on enzymes such as MMP-1 and MMP-9, with relatively few studies on other MMPs (e.g., MMP-3, MMP-12, etc.). Future studies should enhance the standardisation of the chemical composition of the bioactive metabolites and systematically assess their global impact on the balance of MMPs/TIMPs in combination with transcriptomics and proteomics.

#### 4.2.2 Tyrosinase

**FIGURE 2:**
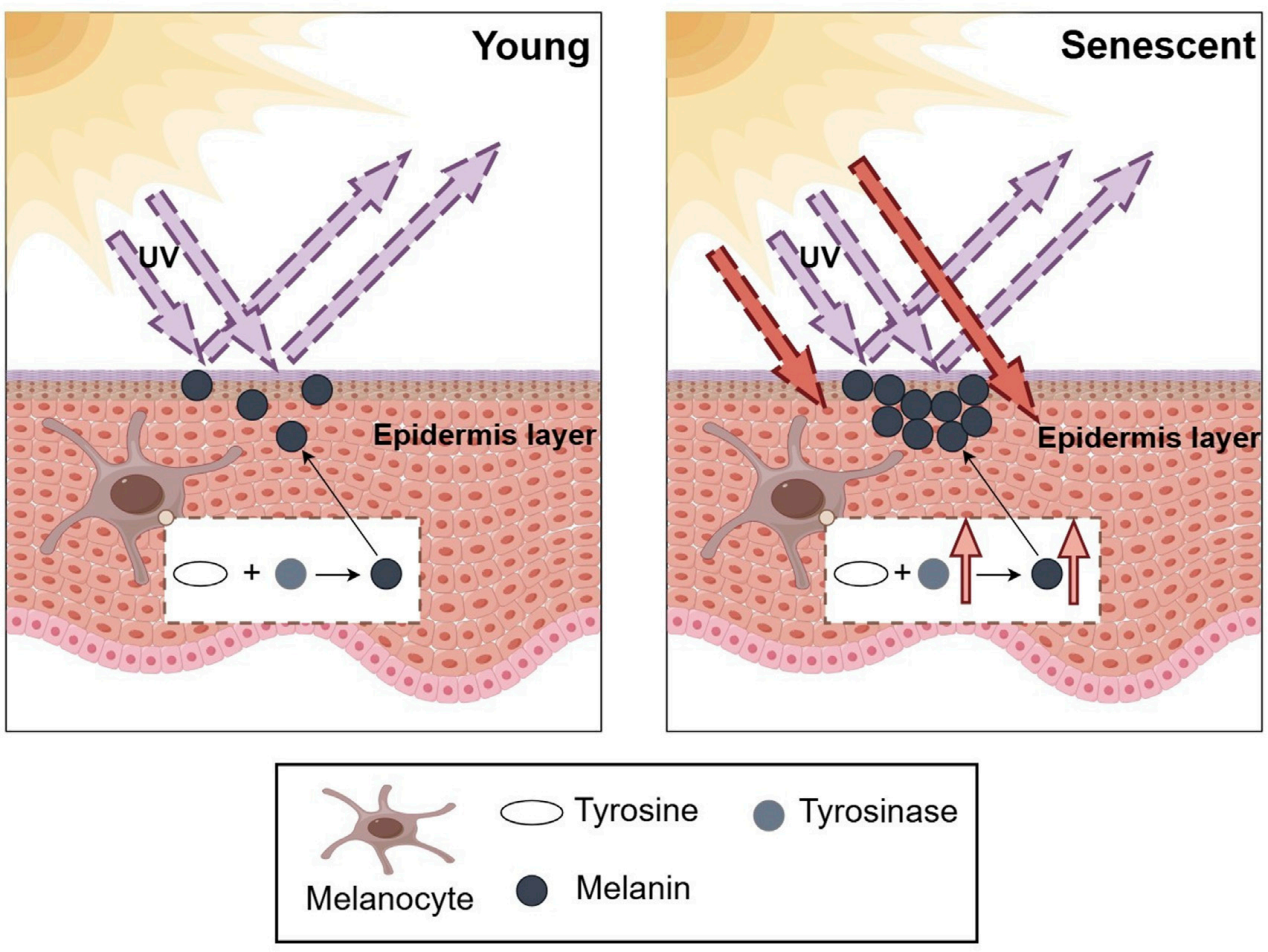
Schematic representation of tyrosinase-mediated skin aging. The increase in tyrosinase activity and melanin synthesis under the effect of increased UV radiation leads to the appearance of uneven pigmentation on the surface of the skin, such as freckles or age spots, a phenomenon that is often regarded as a sign of skin aging.

Tyrosinase plays a key role in oxidative reactions in living organisms (Yuan et al., 2020), catalysing the conversion of Tyrosine into a key precursor of melanin (Nakamura and Fukuda, 2024). Melanin acts as a UV filter, which can effectively absorb and scatter UV radiation, reducing the deleterious effects of UV on the skin (Figure 2). However, the process of melanin synthesis is inherently oxidative, and excess melanin may also lead to senescence of melanocytes, thereby accelerating skin aging (Hughes and Bishop, 2022; Lee, 2021). Botanical bioactive metabolites reduce melanin synthesis by inhibiting tyrosinase activity, thereby improving uneven skin tone and discolouration due to aging.

*Morinda citrifolia* bioactive metabolites, WRPE (116) also inhibit tyrosinase, suggesting that they also have antimelanogenic potential. Litchi chinensis bioactive metabolites has cellular antioxidant activity by reducing melanin production through tyrosinase inhibition (Kanlayavattanakul et al., 2012). Monascus-fermented soybean bioactive metabolites (MFSEs) showed effective inhibition of tyrosinase (P < 0.05) (Jin and Pyo, 2017). A 50% (v/v) EtOH aqueous bioactive metabolites of Rosa gallica petals significantly inhibited tyrosinase activity and reduced melanin production (Shin EJ. et al., 2019). In cellular experiments, cells treated with jasmine rice panicle bioactive metabolites were observed to inhibit melanogenesis via tyrosinase and TRP-2 inhibition (Kanlayavattanakul et al., 2016). H. teretifolium total bioactive metabolites represents a rich source of bioactive metabolites with moderate anti-tyrosinase and anti-elastase activities and thus could be a good candidate for aging prevention (Popoola et al., 2015). Bisresorcinol, a major metabolite of the trunk of Heliciopsis terminalis (Protopanaxaceae), has inhibitory effects on tyrosinase, and bisresorcinol may be used as an aging enzyme antagonist in anti-aging products (Saechan et al., 2021). Solanum betaceum bioactive metabolites (100 μg/mL) inhibited tyrosinase by up to 50.4% (Huang and Huang, 2024). Methanol bioactive metabolites from Mangifera indica leaves (Anacardiaceae) grown in Egypt showed significant anti-tyrosinase effect (El-Nashar et al., 2022). Leaves of Acacia occidentalis and Acacia zeylanica among 16 Thai medicinal plants showed strong antioxidant as well as tyrosinase inhibitory effects. Bioactive metabolites of Amla (Phyllanthus emblica L.) twigs 0.1 mg/mL inhibited melanin by inhibiting tyrosinase and tyrosinase-related protein-2 activities, strong antioxidant properties (Chaikul et al., 2021).

#### 4.2.3 Anti hyaluronidase

Hyaluronidase is an enzyme that breaks down hyaluronic acid. Hyaluronic acid is a key metabolite of the ECM and plays an important role in moisturization, inflammation, cell migration and promotion of collagen synthesis (Gupta et al., 2019). During inflammation, the breakdown of hyaluronan by hyaluronidase leads to the release of pro-inflammatory mediators and chemokines, which exacerbate the inflammatory response. By inhibiting hyaluronidase, plant bioactive metabolites can help maintain hyaluronic acid levels, reduce inflammation and promote tissue repair, highlighting their potential therapeutic applications in inflammatory diseases (HB et al., 2023).

Inflammation is known to upregulate hyaluronidase expression in various tissues, and plant bioactive metabolites can indirectly affect hyaluronidase activity by reducing pro-inflammatory cytokine production (Bonaterra et al., 2020). Trigonella foenum-graecum L. (Eaknai et al., 2022), KTP (Yi et al., 2019), ALE (83), Poria cocos bioactive metabolites (Fang et al., 2021), Vaccinium uliginosum (Jo et al., 2020), AM (Hwang et al., 2018), (−)-phenolic (Lee et al., 2020), Rice (Subedi et al., 2017), Green mandarins (Ham et al., 2022), and DK (Wang et al., 2020) inhibit proinflammatory cytokines such as tumour necrosis factor alpha (TNF-), interleukin, and hyaluronidase. α), interleukin 6 (IL-6), IL-8, etc.) production. In addition, COX-2 mRNA is usually increased during skin inflammation and the Chlamydomonas hedleyi bioactive metabolites Mycosphaerella-Gly also significantly reduced COX-2 mRNA levels in a concentration-dependent manner (Suh et al., 2014).

Plant bioactive metabolites can modulate hyaluronidase activity, thereby affecting the inflammatory response. Evening Thunbergia laurifolia Lindl. leaf bioactive metabolites (Chaiyana et al., 2020), Mangifera indica leaves (El-Nashar et al., 2022), and MFSEs (Jin and Pyo, 2017) have been shown to inhibit hyaluronidase activity, leading to increased hyaluronic acid levels in tissues.In addition, some studies have shown that bioactive metabolites from plants such as marshmallow (Althaea officinalis) (Bonaterra et al., 2020) can lead to a decrease in hyaluronidase expression under inflammatory conditions. This downregulation facilitates the prevention of excessive degradation of hyaluronic acid, thus preserving its protective and moisturising function in tissues.

### 4.3 Modulation of the inflammatory response

Chronic inflammation plays an important role in the skin aging process. Chronic inflammation can lead to a variety of problems in the skin, such as decreased skin barrier function, diminished cell regeneration, and decreased wound healing (Lei et al., 2022). Mediates senescence phenotype by disrupting skin barrier function, attenuating cell regeneration and promoting ECM degradation (Bennett et al., 2008; Agrawal et al., 2023).

#### 4.3.1 Anti-glycation effects

Glycation and the accompanying accumulation of skin advanced glycation end products (AGEs) have been implicated in skin research as one of the mechanisms leading to skin aging (Qu et al., 2017). CF bioactive metabolites reduces AGEs-induced ROS generation and upregulation of the receptor for AGEs (Lyu et al., 2022). JasHEx, which contains phenolic acid derivatives, lignans, and triterpenoids, reduces formation of AGEs (Ceccacci et al., 2022). Air-dried mulberry fruit (DMF) bioactive metabolites was able to reduce oxidative stress by inhibiting glycosylation reactions *in vivo* and avoiding the accumulation of AGEs (Zhang L. et al., 2024). Cirsium japonicum flower (CFE) bioactive metabolites inhibited the formation of AGEs in an *in vitro* glycosylation study (Yoon et al., 2022). In summary, modulation of AGEs by plant bioactive metabolites may be an interesting target for anti-aging.

#### 4.3.2 Phenolic metabolites modulate inflammatory responses

Phenolic metabolites found in plant bioactive metabolites have been shown to modulate inflammatory responses, protect skin cells from damage and promote a healthier appearance (Janaína et al., 2020). *In vitro* studies using cell lines such as RAW 264.7 macrophages have shown that phenolic metabolites in plant bioactive metabolites significantly reduce the production of inflammatory cytokines and mediators when stimulated by lipopolysaccharide (LPS), a common inflammatory trigger (Lee et al., 2021b; Paesa et al., 2022).

One of the key mechanisms by which phenolic metabolites exert their anti-inflammatory effects is through the inhibition of pro-inflammatory mediator production. Inducible nitric oxide synthase (iNOS) and COX-2 are key enzymes involved in the production of inflammatory mediators such as nitric oxide (NO). Reduction of inflammatory mediators reduces inflammation and tissue damage. phenolic metabolites such as bioactive metabolites of TMS-HDMF-5z (a mixture of the natural products mossyloflavone and resveratrol) (Kim et al., 2022) downregulate the expression of iNOS and COX-2. The pro-inflammatory transcription factor NF-kappB plays an important role in the pathology of inflammatory diseases (Lephart, 2016). By inhibiting the translocation of NF-κB to the nucleus, the dried flowers of *C. lanceolata* (Lee et al., 2021b) blocks the expression of several pro-inflammatory cytokines, including TNF-α and IL-6.

## 5 Summary and looking forward

The potential of plant bioactive metabolites to address skin aging offers an attractive option for anti-aging product innovation. This is mainly because they are more in line with the worldwide trend towards naturalness and non-toxicity, and their skincare activity is not inferior to synthetic drugs. Secondly, the study of anti-aging products containing potent plant bioactive metabolites may improve our ability to address future challenges related to skin aging.

Botanical bioactive metabolites are effective in combating skin aging by having antioxidant effects, inhibition of aging-related enzymes, anti-inflammatory properties, and other mechanisms that slow down the aging process and enhance skin health. However, a number of challenges and opportunities remain in the ideal chain of access, research and application of plant skin care metabolites. In terms of access, the anti-skin aging field is progressively prioritising natural products, especially plant bioactive metabolites, a trend that promotes the discovery of anti-aging metabolites from plants. This area of research should focus on plant bioactive metabolites that are photoprotective and mitigate oxidative stress. From a research perspective, it is important to persevere in investigating the specific cellular and molecular mechanisms of action of various plant bioactive metabolites, as this may provide new perspectives to understand their potential efficacy. In addition, the demand for anti-aging products is not limited to preventing skin aging, but also includes daily care. Therefore, research will also focus on the safety and potential side effects of plant bioactive metabolites to ensure that these metabolites can be safely applied in daily care. In the application of plant bioactive metabolites, there is an urgent need to improve the drug delivery method by improving it due to its generally low bioavailability. As an innovative drug delivery strategy, transdermal administration of plant bioactive metabolites, which delivers active metabolites to the body or local target tissues via the dermal route, retains the natural advantages of plant bioactive metabolites while overcoming their inherent limitations through dosage form design. However, it still faces many challenges in terms of skin barrier penetration, stability, and safety. Future research should focus more on advanced transdermal drug delivery systems, such as nanocarrier technologies (including liposomes, nanoemulsions, solid lipid nanoparticles, etc.) and other strategies. These technologies can enhance safety by increasing local bioavailability, improving bioactive metabolites stability, and enabling controlled release. The application of formulations combining multiple plant bioactive metabolites to enhance efficacy requires in-depth research on plant bioactive metabolites and their anti-aging properties. In summary, although plant bioactive metabolites have shown multiple mechanisms and other advantages in anti-skin aging, most of the current studies are still in the preclinical stage, and future research should pay more attention to the completion of high-quality clinical studies, combined with artificial intelligence-assisted screening and other methods, in order to validate the real anti-aging efficacy and safety of these plant bioactive metabolites in the human body, and to promote them to become a scientifically based anti-aging strategy. As research continues to progress and consumers become more aware of the advantages of natural metabolites, the use of plant bioactive metabolites in anti-aging products will make significant progress in the future.
